# Unmet needs in countries participating in the undiagnosed diseases network international: an international survey considering national health care and economic indicators

**DOI:** 10.3389/fpubh.2023.1248260

**Published:** 2023-09-26

**Authors:** Savino Sciascia, Dario Roccatello, Marco Salvatore, Claudio Carta, Laura L. Cellai, Gianluca Ferrari, Aimè Lumaka, Stephen Groft, Yasemin Alanay, Maleeha Azam, Gareth Baynam, Helene Cederroth, Eva Maria Cutiongco-de la Paz, Vajira Harshadeva Weerabaddana Dissanayake, Roberto Giugliani, Claudia Gonzaga-Jauregui, Dineshani Hettiarachchi, Oleg Kvlividze, Guida Landoure, Prince Makay, Béla Melegh, Ugur Ozbek, Ratna Dua Puri, Vanessa I. Romero, Vinod Scaria, Saumya S. Jamuar, Vorasuk Shotelersuk, William A. Gahl, Samuel A. Wiafe, Olaf Bodamer, Manuel Posada, Domenica Taruscio

**Affiliations:** ^1^Center of Excellence on Nephrologic, Rheumatologic and Rare Diseases (ERK-Net, ERN-Reconnect and RITA-ERN Member) With Nephrology and Dialysis Unit, San Giovanni Bosco Hub Hospital, ASL Città di Torino and University of Turin, Turin, Italy; ^2^National Center for Rare Diseases, Undiagnosed Rare Diseases Interdepartmental Unit, Istituto Superiore di Sanità, Rome, Italy; ^3^Reference Center for Rare and Undiagnosed Diseases, University of Kinshasa, Kinshasa, Democratic Republic of Congo; ^4^Service de Génétique Humaine, University Hospitals of Liège, Liège, Belgium; ^5^National Center for Advancing Translational Sciences, National Institutes of Health, Bethesda, MD, United States; ^6^ACURARE-Rare and Undiagnosed Diseases Center, Acibadem University, Istanbul, Türkiye; ^7^COMSATS University Islamabad, Islamabad, Pakistan; ^8^Rare Care, Clinical Center of Expertise for Rare and Undiagnosed Diseases, Perth Children's Hospital, Perth, WA, Australia; ^9^Wilhelm Foundation, Stockholm, Sweden; ^10^Institute of Human Genetics, National Institutes of Health, University of the Philippines Manila, Manila, Philippines; ^11^Department of Anatomy, Genetics and Biomedical Informatics, Faculty of Medicine, University of Colombo, Colombo, Sri Lanka; ^12^House of Rares, Medical Genetics Service, HCPA, Department Genetics UFRGS and DASA, Porto Alegre, Brazil; ^13^International Laboratory for Human Genome Research, Universidad Nacional Autonoma de Mexico, Juriquilla, Queretaro, Mexico; ^14^Georgian Foundation for Genetic and Rare Diseases (GeRaD), School of Medicine, New Vision University, Tbilisi, Georgia; ^15^Faculté de Médecine et d'Odontostomatologie, l'Université des Sciences, des Techniques et des Technologies de Bamako, Bamako, Mali; ^16^Department of Medical Genetics, School of Medicine, University of Pécs, Pécs, Hungary; ^17^Institute of Medical Genetics and Genomics, Sir Ganga Ram Hospital, New Delhi, India; ^18^School of Medicine, Universidad San Francisco de Quito, Quito, Ecuador; ^19^CSIR Institute of Genomics and Integrative Biology, New Delhi, India; ^20^Genetics Service, Department of Pediatrics, KK Women’s and Children’s Hospital and Pediatric ACP, Duke-NUS Medical School, Singapore, Singapore; ^21^SingHealth Duke-NUS Institute of Precision Medicine, Singapore, Singapore; ^22^Center of Excellence for Medical Genomics, Department of Pediatrics, Faculty of Medicine, King Chulalongkorn Memorial Hospital and Chulalongkorn University, Bangkok, Thailand; ^23^National Institutes of Health, National Human Genome Research Institute, Bethesda, MD, United States; ^24^Rare Disease Ghana Initiative, Accra, Ghana; ^25^Division of Genetics and Genomics, Harvard Medical School, Boston Children's Hospital, Boston, MA, United States; ^26^Rare Diseases Research Institute (IIER), SpainUDP, Instituto de Salud Carlos III (ISCIII), Madrid, Spain

**Keywords:** undiagnosed diseases, developing nations, rare diseases, survey, GPD

## Abstract

**Background:**

Patients, families, the healthcare system, and society as a whole are all significantly impacted by rare diseases (RDs). According to various classifications, there are currently up to 9,000 different rare diseases that have been recognized, and new diseases are discovered every month. Although very few people are affected by each uncommon disease individually, millions of people are thought to be impacted globally when all these conditions are considered. Therefore, RDs represent an important public health concern. Although crucial for clinical care, early and correct diagnosis is still difficult to achieve in many nations, especially those with low and middle incomes. Consequently, a sizeable amount of the overall burden of RD is attributable to undiagnosed RD (URD). Existing barriers and policy aspects impacting the care of patients with RD and URD remain to be investigated.

**Methods:**

To identify unmet needs and opportunities for patients with URD, the Developing Nations Working Group of the Undiagnosed Diseases Network International (DNWG-UDNI) conducted a survey among its members, who were from 20 different nations. The survey used a mix of multiple choice and dedicated open questions covering a variety of topics. To explore reported needs and analyze them in relation to national healthcare economical aspects, publicly available data on (a) World Bank ranking; (b) Current health expenditure *per capita*; (c) GDP *per capita*; (d) Domestic general government health expenditure (% of GDP); and (e) Life expectancy at birth, total (years) were incorporated in our study.

**Results:**

This study provides an in-depth evaluation of the unmet needs for 20 countries: low-income (3), middle-income (10), and high-income (7). When analyzing reported unmet needs, almost all countries (*N* = 19) indicated that major barriers still exist when attempting to improve the care of patients with UR and/or URD; most countries report unmet needs related to the availability of specialized care and dedicated facilities. However, while the countries ranked as low income by the World Bank showed the highest prevalence of referred unmet needs across the different domains, no specific trend appeared when comparing the high, upper, and low-middle income nations. No overt trend was observed when separating countries by current health expenditure *per capita*, GDP *per capita*, domestic general government health expenditure (% of GDP) and life expectancy at birth, total (years). Conversely, both the GDP and domestic general government health expenditure for each country impacted the presence of ongoing research.

**Conclusion:**

We found that policy characteristics varied greatly with the type of health system and country. No overall pattern in terms of referral for unmet needs when separating countries by main economic or health indicators were observed. Our findings highlight the importance of identifying actionable points (e.g., implemented orphan drug acts or registries where not available) in order to improve the care and diagnosis of RDs and URDs on a global scale.

## Introduction

An accurate diagnosis is the cornerstone of medicine; it is essential for informed care and promoting patient and family well-being. However, individuals and families living with a rare disease (RD) typically spend more than 5 years on a diagnostic odyssey of specialist visits and invasive testing that is lengthy, costly, and often futile. Graessner and colleagues estimated that up to 50% of patients with a rare disease remain undiagnosed even in advanced expert clinical settings where genome sequencing techniques are applied routinely (1). The main reason is that the current diagnostic paradigm based on single-center expertise is not well designed for rare diseases, especially for patients who remain undiagnosed after the initial set of investigations (clinical, instrumental, and laboratory). In this setting, collaboration among all parties (clinicians, researchers, and patient organizations) is essential to reduce the burden associated with a delayed diagnosis and improve the overall management of patients living with rare diseases.

Among ongoing national and international initiatives, the Undiagnosed Diseases Network International (UDNI) (2), by actively involving patients and patient organizations, aims to reduce or eliminate information gaps that contribute to delay diagnosis and promote the integration of research into clinical practice in a process of mutual education.

To map unmet needs and opportunities, a first pilot survey was launched in October 2020 among the UDNI Developing Nations Working Group (UDNI DN WG, hereafter WG) (3). The WG is composed of representatives from 20 countries, including developed as well as low- and middle-income countries (LMIC). In this work, we present the findings of a follow-up survey, aimed to better characterize the global scenario, focusing on existing barriers and policy aspects impacting the care of patients with rare and undiagnosed diseases.

## Methods

### Survey design

Based on the results of the pilot survey (3), this study was developed within the WG to further investigate the main needs for diagnosing undiagnosed patients, aiming to better characterize existing barriers, explore policy aspects, and correlate those needs to national healthcare economics and advocacy organizations.

The survey was structured into three domains, each explored through a mix of multiple choice and dedicated open questions. The results were structured based upon descriptions provided by each country’s representative and summarized in tables and figures. Three domains were addressed.

Domain 1. Unmet needs.
Domain 2. Characteristics of healthcare organizations.
Domain 3. Regulatory aspects and policy.

The survey’s technical functionality and overall consistency were first tested among the co-chairs of the Working Group and members of their respective teams. The WG representatives from all 20 nations were then informed of the WG objectives and supported them. This approach has been previously detailed (3).

### National health care and economic indicators

To explore reported needs and analyze them in relation to national healthcare economical aspects, publicly available data on the following indicators were retrieved and incorporated in our study: (a) World Bank ranking; (b) Current health expenditure *per capita*; (c) GDP *per capita*; (d) Domestic general government health expenditure (% of GDP); and (e) Life expectancy at birth, total (years). Categorization into quartiles for each indicator were used for our analyses. Data were retrieved as open source in the World Health Organization Global Health Expenditure database.

The Global Health Expenditure Database provides comparable data on health expenditure for more than 190 WHO Member States since 2000 with open access to the public. WHO works collaboratively with Member States to update the database annually, using available information such as health accounts data, government expenditure records, and official statistics. The database represents a unique data source to perform research on public health topics, such as health care functions, primary health care, spending by diseases, and conditions (apps.who.int./nha/database, Accessed August 21, 2023).

## Results

Undiagnosed Diseases Network International members of the 20 countries involved in this study (100%) provided their responses based on their own knowledge of the country’s situation on a comprehensive array of RD-related challenges. Each participant member was actively involved in RD at a high level in their own nation. Each respondent answered the questions based on his or her specific understanding of the country’s RD ecosystem and URD condition.

The main characteristics of the participant members, including the type of institution, the presence of an initiative dedicated to undiagnosed and rare diseases within the country’s health care system, the presence of a specific genetic program dedicated to undiagnosed and rare disease, and the type of coverage are described in Table 1. The UDNI countries and country representatives contributing to the UDNI Developing Nations Working Group have been previously described (3). In this study, we provide descriptors to explore the current scenario of rare diseases across different countries with a specific focus on policies in place.

**Table 1:** Main characteristics of available infrastructures for undiagnosed and rare diseases.

Country	Type of institutions	Presence of a dedicated clinical program for undiagnosed and rare diseases within the country’s health care system (name, year of foundation)	Presence of a specific genetic program dedicated to undiagnosed and rare diseases (name, year of foundation)	Type of coverage
Australia	Public Hospital; Designated Center for Rare Diseases; Research, National Undiagnosed Diseases Network (UDN-Aus)	Healthcare system in Western Australia (2016, Undiagnosed Diseases Program-WA)	Healthcare system in Western Australia (Undiagnosed Diseases Program-WA, 2016)	Initiated with philanthropy and now funded by Western Australian State Health System; Research, (UDN-Aus) limited funding by national research funding body
Brazil	Center dedicated to diagnosis, care, and training in rare diseases	Undiagnosed Disease Program promoted by “Casa dos Raros,” to start in 2023 (when the facility became operational)	Planned	Partially covered by the National Health System (SUS)
Congo	Research Center; Academic Hospital; Designated Center for Rare Diseases	Center For Rare And Undiagnosed Diseases (CRMRND) created in 2022	CRMND and the Center for human genetics in Collaborations with international consortia are ongoing	Patients (their families) pay for everything except for genetic testing
Ecuador	Private university	-	-	Private insurance
Georgia	Research Center; Designated Center for Rare Diseases; Academic Hospital; NGO uniting people personally affected by rare diseases (medical professionals, researchers, and patients), working in the field of RD; and Patients Representative Association	(1) The Unified State Program on Rare Diseases (treatment issues)	-	Partially covered by the National Health System
		(2) The Program on outpatients surveillance of PLWRD (not include genetic testing)		
		(3) City Halls (municipal) based treatment program on RD patients (e.g., medical nutrition); State Referral Program (full or partial financing of the diagnosis or treatment in selected cases)		
		(4) State or Municipal based rehabilitation programs (e.g., neuromuscular diseases)		
Ghana	Patients Representative Association; Public Hospital	- RDGI Diagnostic Access program - Newborn Screening by the Sickle Cell Foundation Ghana - Genetics Training Program at the West Africa Genetics Medicine Center	The RDGI Diagnostic Access Program provides genetic diagnosis to patients suspected for a rare disease. The West Africa Genetic Medicine Center is currently training geneticist and genetic counselors in the country	Cost covered by no profit organization
Hungary	Academic Hospital; Public Hospital; Diagnostic Facility (GENOMIC); Research Center; Designated Center for Rare Diseases; and Public Research Institute	Not on a National Level. Participation in ERN projects, specific research projects by national health authorities and by research institutions	Planned	Partially covered by the National Health System
India	Academic Hospital Trust Private Hospital; Research Center; Designated Center for Rare Diseases	Indian Undiagnosed Diseases Program (I-UDP)	Indian Undiagnosed Diseases Program (I-UDP)	Combination of Healthcare system and Out of pocket expenditure
		By other initiatives	By other initiatives	
		3 years	3 years	Partially covered by the National Health System
		GUaRDIAN—Genomics for Understanding Rare Diseases India Alliance Network (http://guardian.genomes.in; 2015-2025)	GUaRDIAN—Genomics for Understanding Rare Diseases India Alliance Network (http://guardian.genomes.in; 2015-2025)	
Italy	Public Research Institute	UDP-Italy, since 2016 up to now: 7 years	UDP-Italy activated in 2016 and it is running up to now: 7 years	Partially covered by the National Health System
Mali	Academic Hospital; Public Hospital; Public Research Institute	-	-	The research programs listed above cover partially for enrolled patients
Mexico	Public Research Institute; Research Center; Public University	Not on a National Level or only on selected topics [e.g., national newborn screening program (healthcare system), expanded newborn screening program in certain public and private hospitals, research program in undiagnosed, and rare diseases (public university) not related to healthcare system (1.5 years)]	Research program for Undiagnosed and Rare Diseases started in 2022 in National Public University, other disease-specific efforts in different public hospitals and institutions	Some covered by the healthcare system, most covered by patients out-of-pocket or with help from patient organizations
				Genetic diagnosis academic program covered through research, donations and out-of-pocket patient contributions
Pakistan	Public Research Institute	None	Only at medical and research institutional level	Research grants to individual researchers and Self-financed by the families
Philippines	Academic Hospital; Research Center	Department of Health	Integrated Rare Disease Management Program of the Department of Health	Out of pocket
		Philippine Society for Orphan Disorders	Philippine Society for Orphan Disorders (Patient Support Group)	
Singapore	Academic Hospital	BRIDGES: Bringing Research Innovations for Diagnosis of GEnetic diseases in Singapore, since 2014. By KK Women’s and Children’s Hospital. Collaboration with National University Hospital	BRIDGES: Bringing Research Innovations for Diagnosis of GEnetic diseases in Singapore, since 2014. By KK Women’s and Children’s Hospital. Collaboration with National University Hospital	Research grants/Philanthropy
Spain	National Center for Rare Diseases; Public Rare Diseases Research Institute; Diagnostic Facilities including GENOMIC and functional studies	SpaiunUDP. http://spainudp.isciii.es 2015	SpaiunUDP. http://spainudp.isciii.es 2015	Covered by the Institute of health Carlos III (SCIII). Dedicated annual Budget managed by the Rare Diseases Research Institute, ISCIII
Sri Lanka	Academic Hospital	Rare Disorders Forum dedicated to sharing information hosted by the Sri Lanka College of Pediatricians.	Only at institutional level	Partially covered by the National Health System
Sweden	Wilhelm Foundation	Karolinska UDP, healthcare and research, 2018-CSD Centrum för Sällsynta Diagnoser, healthcare system, ongoing in all six university hospitals	Karolinska UDP, healthcare and research, 2018	Fully covered by the National Health System
Thailand	Academic Hospital; Public Hospital; Diagnostic Facility (GENOMIC); Research Center	(1) “24 rare diseases under the Universal Healthcare Scheme,” foreseen by the healthcare system, started in the year 2000 onwards	(1) “24 rare diseases under the Universal Healthcare Scheme,” foreseen by the healthcare system, covers the genetic and biochemical tests	Partially covered by the National Health System
		(2) “Rare disease—whole genome sequencing under Genomics Thailand,” foreseen by Health Systems Research Institute, during 2019–2024	(2) “Rare disease—whole genome sequencing under Genomics Thailand”	
Turkey	Academic Hospital; Diagnostic and Treatment Clinics for Rare Diseases; Research Center	Not on a National Level. Participation in EU and International projects, specific research projects by national health authorities and by research institutions	Istanbul Rare and Undiagnosed Solution Platform—ISTISNA project started by 2022 in connection with UDNI diagnostics group. Advisory board, genomic sequencing and data reanalyzes are major contributions	Partially covered by the National Health System
United States	Government research center—NIH United States	NIH-supported Undiagnosed Diseases Network 2014–2023	A consortium of Undiagnosed Diseases Programs, with modest NIH funding, is planned for the country. Genetic sequencing and analyses will be a major part of that consortium	Private insurance
		Non-government-supported programs at some medical centers such as Birmingham, Mayo, etc.		
		NIH Intramural Research Program has funding from 2023–2028		

We collected data on the existence of National Rare Disease Policy dedicated to undiagnosed and/or rare diseases, Orphan Drug Acts, National Registries dedicated to undiagnosed and/or rare diseases, and Patient Association programs (Table 2). To improve comparability, the definition of rare disease for each country is also provided. As expected, the organization of health care for RDs and the presence of programs and activities in place vary considerably across countries (Table 3).

**Table 2:** Policy aspects related to rare disease.

Country	National Rare Disease Policy dedicated to undiagnosed and/or rare diseases	Is there an orphan drug act in your country	National Registry dedicated to undiagnosed and/or rare diseases	Definition of rare diseases	National Rare Disease Patient Organization program dedicated to undiagnosed and/or rare diseases
Australia	Plan for Rare Diseases, The Action Plan aligns with, and expands on, the Call for a National Rare Disease Framework: six Strategic Priorities, which was published by RVA in June 2017.	The Therapeutic Goods Administration (TGA) Orphan Drugs Program.	No	No national consensus	Rare Voices Australia (RVA) was established in 2012
		An amendment to the Therapeutic Goods Act 1989 was made in 1997 to include orphan drugs under section 16H.			
Brazil	*National Policy for Integral Attention to People with Rare Diseases* (Resolution 199/2014—Ministry of Health—Brazil)	Resolution 563/2017 of the National Health Council, which establishes guidelines for the evaluation of clinical trials involving rare diseases in Brazil; and ANVISA resolutions RDC 205/2017, RDC 505/2021, RDC 506/2021 and RDC 508/2021, which establish a framework for the evaluation of advanced therapies for rare diseases by the sanitary authorities in Brazil.	Not formally established, with some pilot initiatives in progress	Not more than 65 cases for each 100,000 individuals	Febrararas (Brazilian Federation of Rare Disease Associations)
Congo	No	No	No	No local definition exists. European definition (<1/2000) is widely used	No
Ecuador	No	Any health condition treatment is free of charge legally in Ecuador. However, it is not true. Patients have to get the approval of many doctors to receive the treatment, and sometimes it is too late.	No	No local definition exists. Chronic and disabling aspects are considered	No
Georgia	No	No	No	The definition is the same as that adopted in the EU countries (Proposed by the Georgian Foundation for Genetic and Rare Diseases in 2014)	No
Ghana	No	FDA guidelines for registration of orphan drugs	Congenital anomalies and rare disease registry	Diseases that are not common	Rare Disease Ghana Initiative
Hungary	No national plans. At Institutional level.	No	No	EU definition	RIROSZ
India	https://main.mohfw.gov.in/sites/default/files/Final%20NPRD%2C%202021.pdf	No	National Registry for Rare Diseases has been initiated by ICMR	No local definition exists.	https://ordindia.in/
	https://rdrdb.icmr.org.in/				https://www.rarediseases.in/
					Both these organizations are dedicated to rare diseases
Italy	Italian Law 17/2021	Regulation (EC) N. 141/2000 of the European Parliament and of the Council of 16 December 1999 on orphan medicinal products	National Registry for Rare Diseases	Five cases in 10,000 citizens (EU definition)	Hope, Malati Invisibili, UNIAMO
Mali	No	No	No	No local definition exists	No
Mexico	Ley General de Salud (Art. 224 Bis) addresses the definition for rare diseases in Mexico in the context of access to orphan drugs	Ley General de Salud (Art. 224 Bis) addresses access to orphan drugs but no complete legislation	Yes, since 2022	No more than 5 in 10,000	Three main patient umbrella organizations for RDs in the country
Pakistan	No	No	No	No local definition exists	No
Philippines	Republic Act No. 10747 Rare Disease Law	The Integrated Rare Diseases Management Program addresses this as one of its strategic objectives, i.e., increase availability and access to orphan drugs and products	A multi-sectoral strategic plan for the integrated rare diseases management program for the period 2022–2026 has been developed and is being implemented by the Department of Health	One in 20,000	Planned
	An Act promulgating a comprehensive policy in addressing the needs of persons with rare disease				
Singapore	No	No	Singapore Genetics Registry	Disease affecting less than 1 in 2000 individuals	Rare Disease Society of Singapore
Spain	-	No	There is a national RD patient registry https://registroraras.isciii.es. This registry includes the undiagnosed cases belonging from the SpainUDP program	The same than in EU less than five cases per 10,000	Multiple partners (e.g., FEDER; D’GENES; Objetivo Diagnostico)
Sri Lanka	Although there is no policy, patients with any rare disorder is entitled to receive free access to care in the national health service	No	No	No country specific definition. United Kingdom definition usually applied	No
Sweden	No	No	No	In Sweden, it is called Rare Diagnosis and prevalence is less than 5/10000	Sällsynta Diagnoser, and Wilhelm Foundation
Thailand	Yes. Since 2019, Thailand’s National Health Security Office has included 24 rare diseases into the universal health coverage scheme	The Thai FDA has defined an “orphan drug” as: (1) A drug needed for diagnosis, alleviation, treatment, prevention, or cure of: a rare disease, a seriously harmful disease, or a disease resulting in continuing disabilities. (2) A drug with a low usage rate, wherein there is no other drug that can be utilized as a replacement, and there is also a shortage issue	Rare disease area of Genomics Thailand	Fewer than 10,000 affected individuals in the country	Rare Disease Patients Network, which has been established for decades by a family with a rare inborn error of metabolism
Turkey	National Rare Diseases Action Plan, Ministry of Health, 2023	National Rare Diseases Action Plan refers, although no orphan medicinal products legislation yet.	No	1/2000	Rare Diseases Network, Turkey
United States	Rare Disease Act of 1983	Rare Disease Act of 1983 provides incentives for development of orphan drugs.	No	One that affects fewer than 200,000 Americans	NORD, Genetic Alliance

**Table 3:** Main reported needs for undiagnosed and rare diseases.

Access to care	Availability of specialized care	Availability of dedicated facility	Limited ongoing research	Other
Australia	Yes	Yes	Yes	Policy changes at decision making level
Brazil	Yes			-
Congo	Yes	Yes	Yes	No RD policy
Ecuador	Yes		Yes	Access to genetic testing and funding
Georgia	Yes	Yes	Yes	-
Ghana	Yes	Yes	Yes	-
Hungary	Yes	Yes	Yes	-
India	Yes	Yes	Yes	-
India	Yes	Yes	Yes	
Italy		Yes		-
Mali	Yes	Yes	Yes	-
Mexico	Yes	Yes	Yes	Access to genetic testing and funding
Pakistan	Limited	No	Yes	No RD policy
Philippines	Yes	Yes		Access to genetic testing and funding
Singapore	Yes	Yes	Yes	Funding
Spain			Yes	Access to genetic testing and funding
Sri Lanka	Yes	Yes	Yes	Access to genetic testing and funding
Sweden	Yes	Yes		
Thailand	Yes	Yes	Yes	
Turkey	Yes	Yes	Yes	-
USA				-

**Figure 1:**
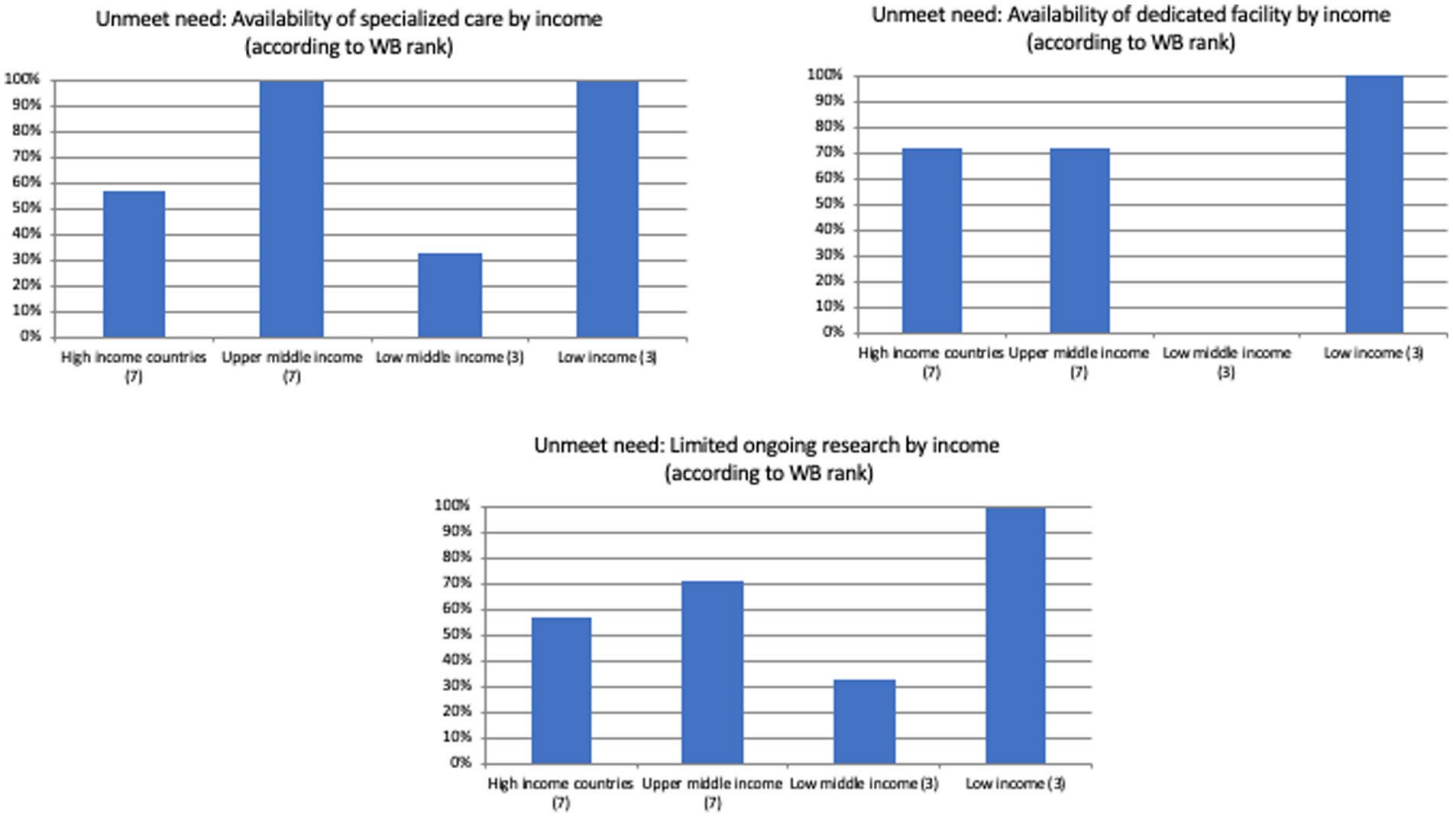
Unmet needs to tackle undiagnosed patients in different countries divided by the World Bank ranking.

When analyzing reported unmet needs, almost all countries (*N* = 19) indicated that major barriers still exist when attempting to improve the care of patients with rare and/or undiagnosed diseases; most countries report unmet needs related to the availability of specialized care and dedicated facilities (Table 3). Figure 1 shows the unmet needs to tackle undiagnosed patients in different countries divided by World Bank classifications. While the countries ranked as low income by the World Bank showed the highest prevalence of referred unmet needs across the different domains, no specific trend appeared when comparing the high, upper, and low-middle income nations.

**Figure 2:**
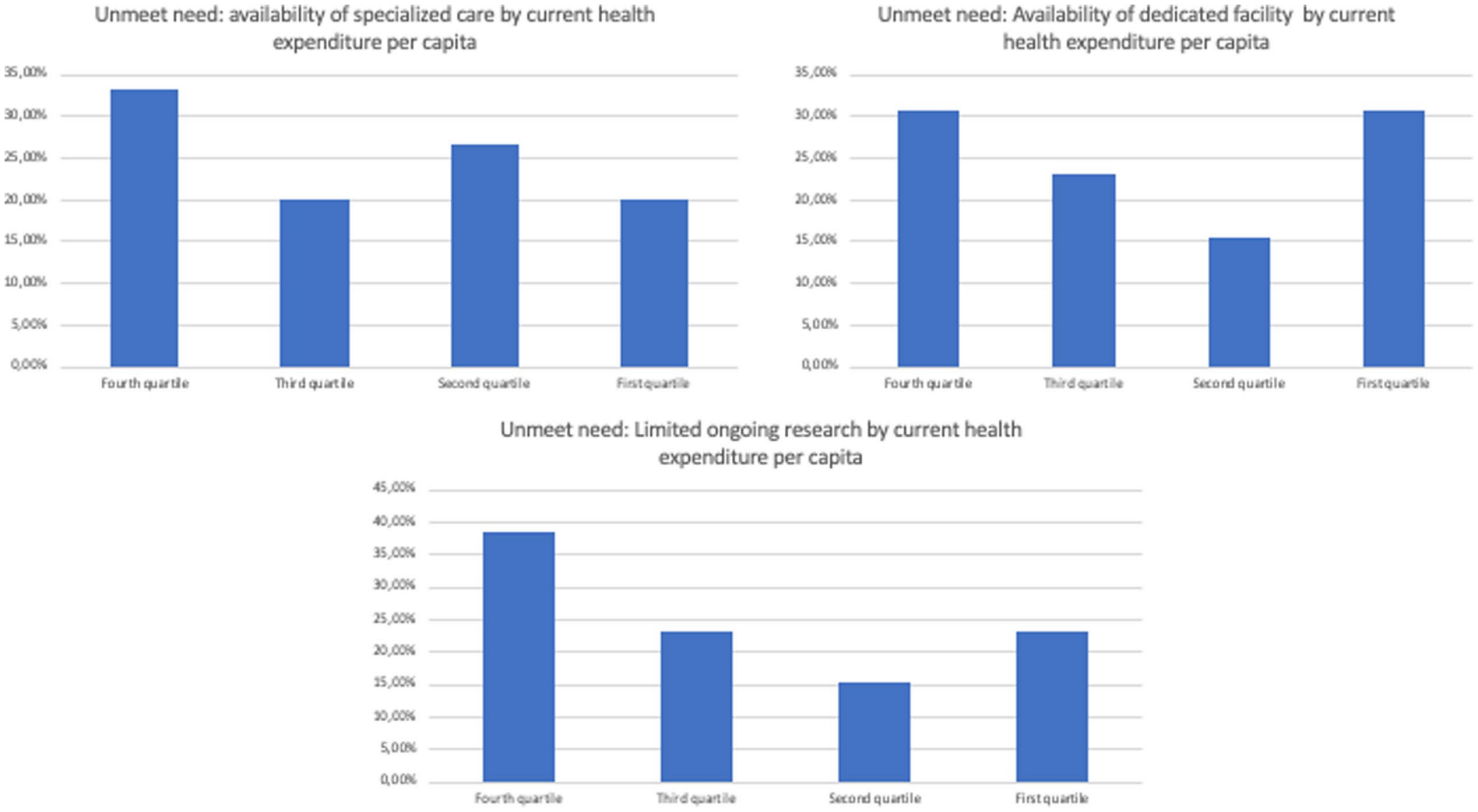
Unmet needs to tackle undiagnosed patients in different countries divided by the current health expenditure *per capita*.

**Figure 3:**
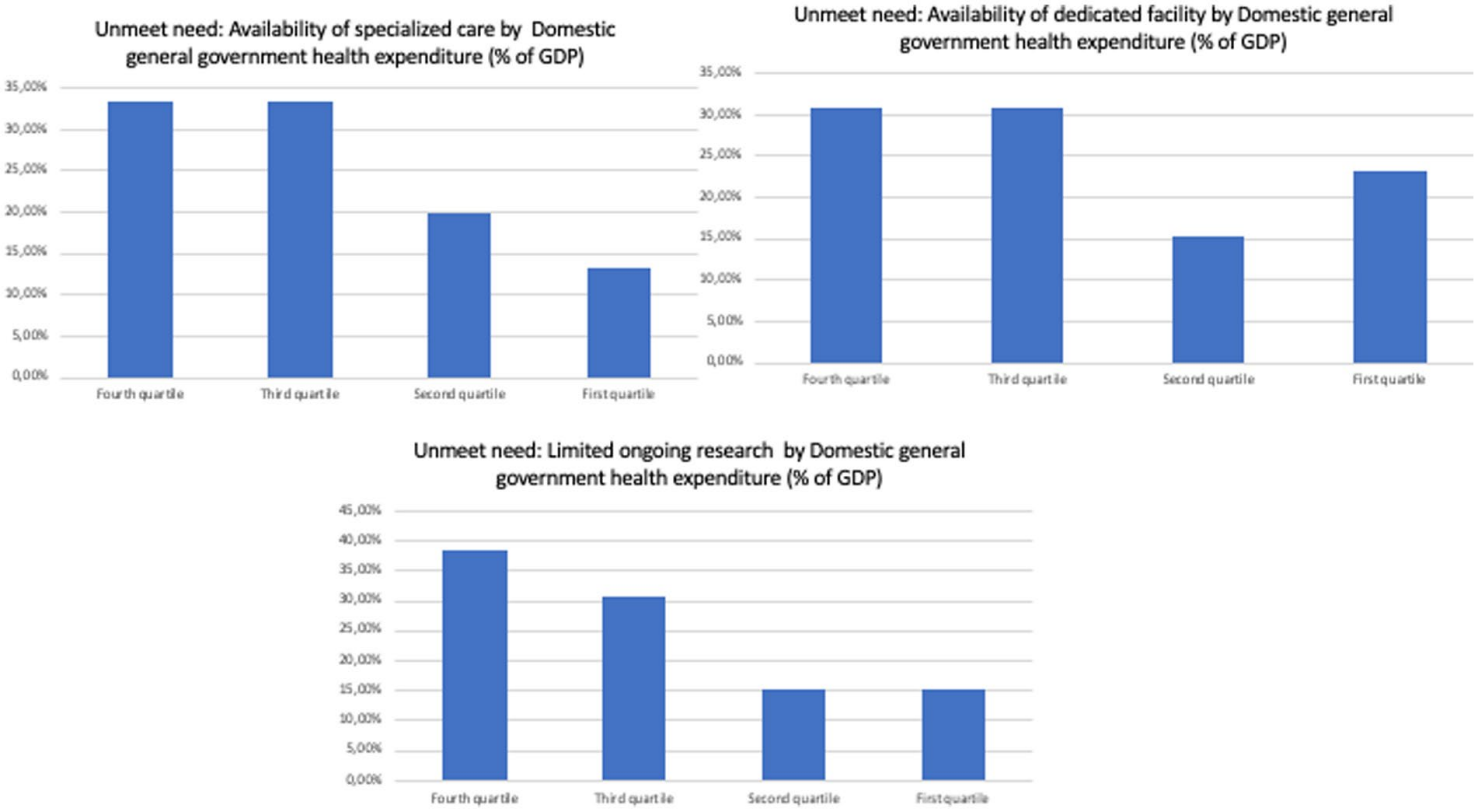
Unmet needs to tackle undiagnosed patients in different countries divided by domestic general government health expenditure (% of GPD).

**Figure 4:**
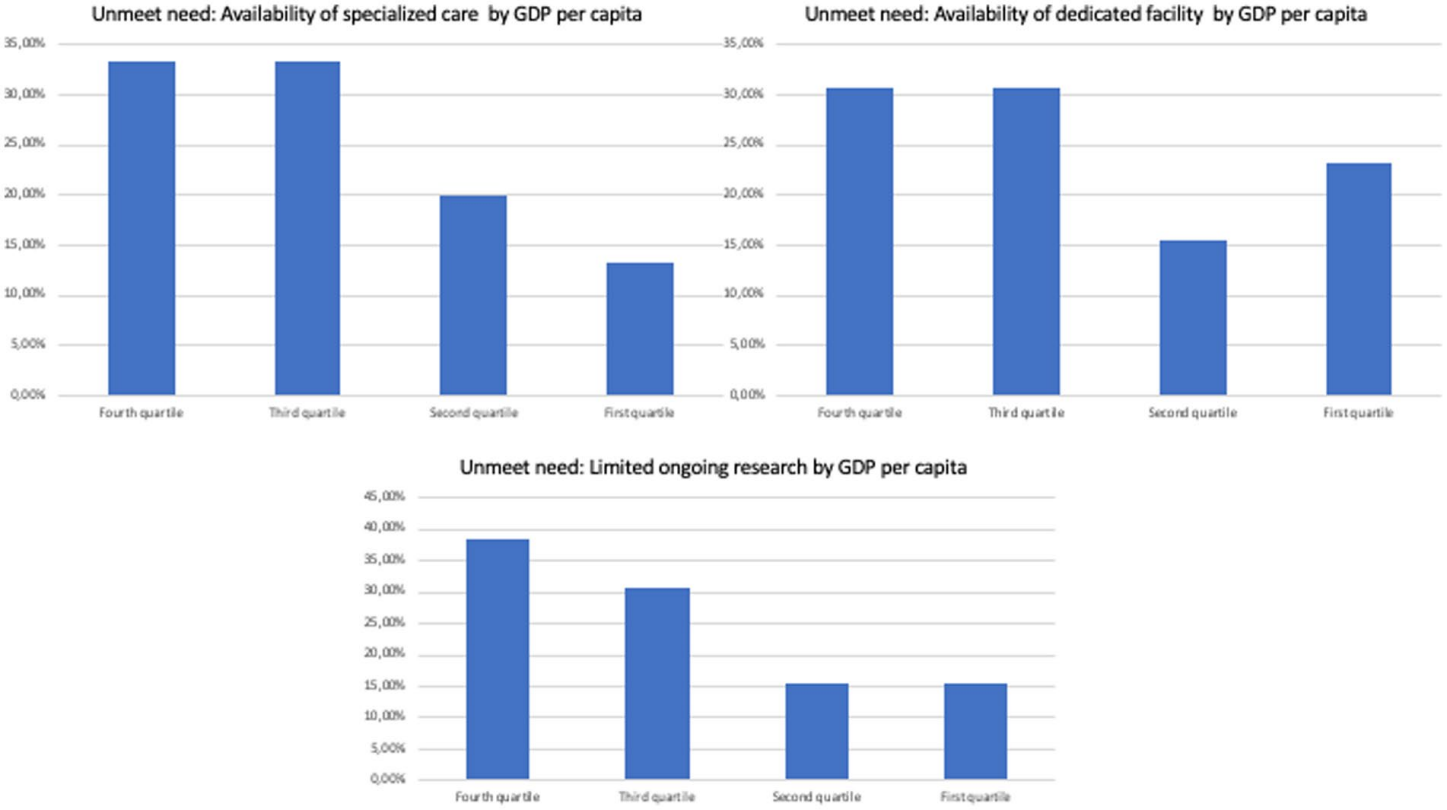
Unmet needs to tackle undiagnosed patients in different countries divided by GPD *per capita*.

**Figure 5:**
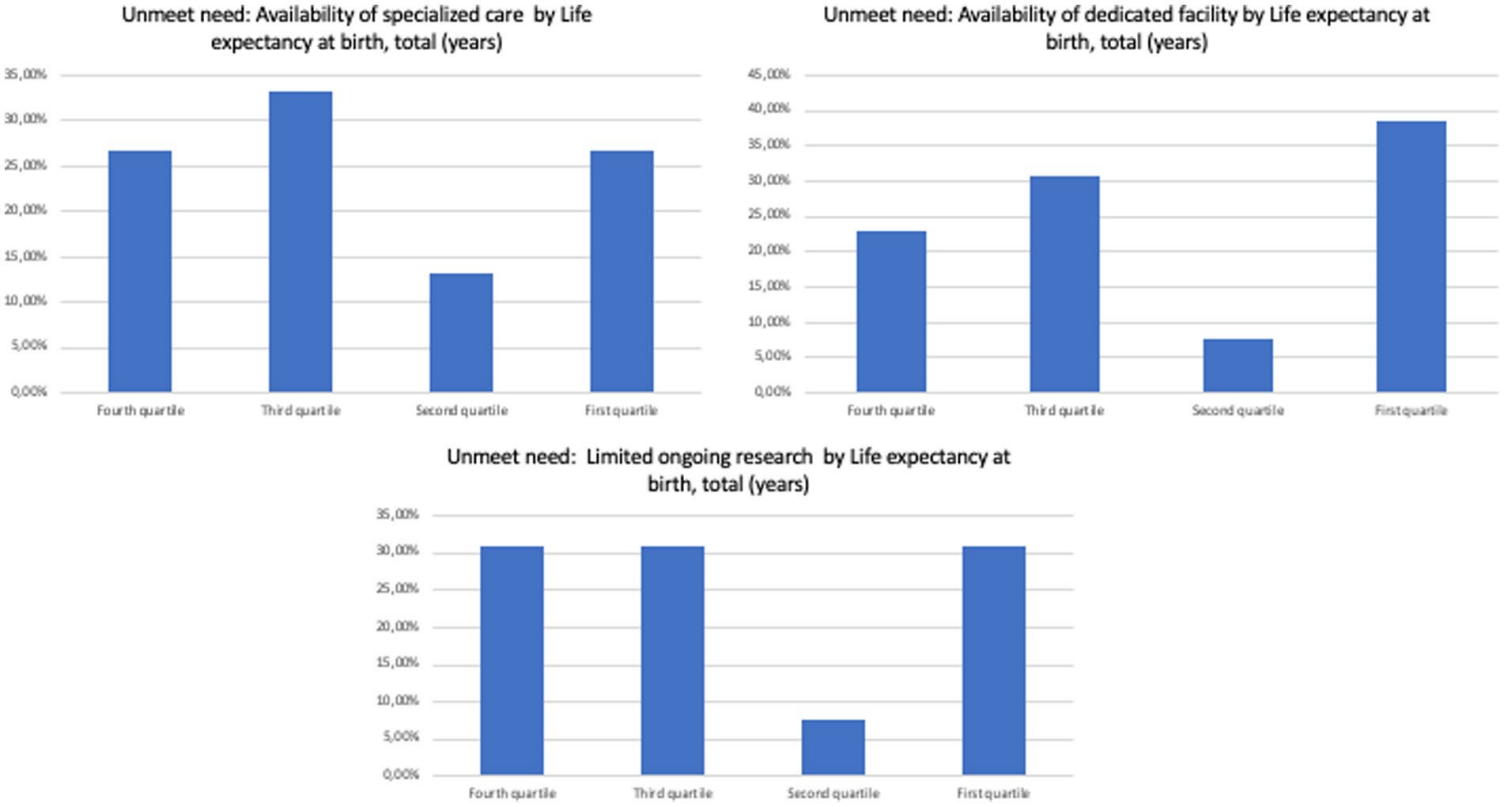
Unmet needs to tackle undiagnosed patients in different countries divided by life expectancy at birth.

Specifically, no overt trend was observed when separating countries by current health expenditure *per capita*, GDP *per capita*, domestic general government health expenditure (% of GDP), and life expectancy at birth, total (years; Figures 2–5). However, both the GDP and domestic general government health expenditure for each country impacted the presence of ongoing research in the field of rare and undiagnosed diseases.

## Discussion

This study provides an in-depth evaluation of the unmet needs for 20 countries: low-income (3), middle-income (4), and high-income (5). We found that policy characteristics varied greatly with the type of health system and country. Interestingly, we observed no overall pattern in terms of referral for unmet needs when separating countries by main economic or health indicators were observed. Our analysis is one of the first in its kind, especially when attempting to explore trends in national health care and economic indicators through the lens of URDs across different countries. While the possibility of comparisons with previous studies is limited, the lack of overt trends between the selected indicators and the main reported unmet needs requires further investigations. Indicators such as current health expenditure *per capita*, GDP *per capita*, and domestic general government health expenditure (% of GDP) have been associated to trends in health outcomes (e.g., Mortality-to-Incidence Ratio) in different settings, including oncologic and non-communicable diseases (5–8). Conversely, our analysis shows a general agreement in terms of actions needed to foster the care of patients with RD and URDs. This observation supports the concept that the global challenge for the future of rare and undiagnosed diseases includes the identification of actionable points (e.g., implemented orphan drug acts or registries where not available) and this perception is shared across the members of the UNDI participating in this survey.

On the other hand, our analysis suggested that more interest in research in the field in RDs and URDs is reported in countries with higher income. These trends are aligned with those observed in medical research beyond the field of RD, suggesting that higher GDP or government health expenditure might correlate with more medical research activity when evaluated through different outcome measures (e.g., number of published papers, R&D expenditure by sector) (4, 9–12).

Some limitations of our survey include the limited sample size and lack of the ability to perform inference analysis. Similarly, we note that participants in the survey were selected from among members of the UDNI. Further research effort is currently ongoing to expand these aspects.

It should also be considered that, as there are no universal epidemiological criteria for RDs (13), the concept of RD in the current political and legislative framework is closely linked to a definition that may vary across countries. When assessing the population burden of disease, point prevalence is probably the most appropriate indicator for RDs. However, for the purpose of our study, while we cannot exclude a degree of heterogeneity in the applied definitions of RDs in the countries participating in our survey as shown in Table 2, it seems unlikely that these differences might have significantly impacted our observations due to the macrolevel approach of our analysis.

Our analysis also highlighted the heterogeneity in terms of policy for RDs and URDs across countries. A National Rare Disease Policy dedicated to undiagnosed and/or rare diseases is in place in nine out of the 20 countries participating to our survey, an orphan drug act in nine out of 20 and only eight registries have been implemented at national level, albeit with very different characteristics. Conversely, at least half of the participating countries report the presence of one or more patients’ associations supporting people with RDs.

The diagnostic odyssey for rare and undiagnosed disease patients must be shortened, their management must be improved, the morbidity and early mortality of these patients must be decreased, and the socioeconomic potential and quality of life of these patients must all be improved by joint national and international initiatives.

## Data availability statement

The raw data supporting the conclusions of this article will be made available by the authors, without undue reservation.

## Author contributions

SS, DR, DT, MS, CC, and LC analyzed the data and were the major contributor in drafting the manuscript. GF, SG, YA, MA, GB, HC, EC, VD, RG, CG-J, DH, OK, GL, PM, BM, UO, RP, VR, VSc, SJ, VSh, WG, SW, OB, AL, and MP carefully revised draft, tables, and figures, and approved final manuscript. All authors contributed to the article and approved the submitted version.
